# Religious Fasting Following Metabolic and Bariatric Surgery (MBS): Insights from Jewish Practices in Israel

**DOI:** 10.3390/medicina60122058

**Published:** 2024-12-13

**Authors:** Shai Meron Eldar, Andrei Keidar, Adam Abu-Abeid, on behalf of the ISMBS Collaborative Group

**Affiliations:** 1 Division of General Surgery, Tel Aviv Sourasky Medical Center, Tel-Aviv 6423906, Israel; ndrkdr@gmail.com (A.K.); adama@tlvmc.gov.il (A.A.-A.); 2 The Faculty of Medical and Health Science, Tel-Aviv University, Tel-Aviv 69978, Israel

**Keywords:** bariatric surgery, complications, religious fasting, expert opinion, survey

## Abstract

*Background and Objectives*: Religious fasting in patients after Metabolic and Bariatric Surgery (MBS) remains a topic with limited clarity. This study aims to present the results of a survey on religious fasting in patients after MBS in Israel. The questionnaire was sent to members of the Israeli Society for Metabolic and Bariatric Surgery (ISMBS). *Materials and Methods*: An online questionnaire survey was designed and distributed to members of the ISMBS. The survey consisted of 23 questions addressing religious fasting in patients after MBS and was divided into three sections: (1) MBS surgeon clinical experience, (2) clinical considerations regarding religious fasting in MBS patients, and (3) fasting-related complications in MBS patients. Responses were recorded and presented as numbers (percentages), with results analyzed descriptively and/or graphically. *Results*: The ISMBS has 63 active members, and 37 members (59%) responded to the survey. Most respondents have more than 10 years of MBS experience and perform more than 100 MBS procedures annually (67.5% and 54%, respectively). In general, 81.1% of respondents permit religious fasting in patients after MBS, and 73% think that fasting could be safe at least 12 months after MBS. Most (62.2%) agree that a clinical evaluation should be undertaken prior to permitting religious fasting; 40% of respondents note that there is increased patient admission to emergency rooms during religious fasting, mostly due to dehydration. When asked about fasting risks, most noted hypoglycemia (40.5%) and the evolution of marginal ulcers (16.2%). *Conclusions*: In conclusion, these national survey results emphasize the variations in MBS surgeons’ opinions regarding religious fasting after MBS. Despite these differences, there were still many similarities in responses such as timing and fasting permission, and this study could aid clinicians in the future when consulted on religious fasting by MBS patients.

## 1. Introduction

Fasting is a common religious practice observed across various faiths, including Islam, Christianity, Buddhism, Hinduism, and Judaism [1]. Patients often seek guidance from physicians about whether they can safely fast during religious holidays. Fasting may carry health risks for patients with chronic illnesses and could potentially exacerbate their condition. Therefore, an individualized case by case approach is essential, and each patient’s circumstances should be carefully evaluated when they enquire about fasting [2].

Metabolic and Bariatric Surgery (MBS) is the most effective treatment for patients with severe obesity [3]. Both observational and prospective studies have shown favorable outcomes, including sustained long term weight loss, the resolution of obesity-mediated medical conditions, and enhanced quality of life [4,5,6]. During follow-up, MBS patients are regularly screened for potential chronic complications, and when necessary, referred for further evaluation [7].

The impact of religious fasting on MBS patients is a significant area of interest. MBS procedures typically alter the anatomy and function of the gastrointestinal tract, which can increase the risk of nutritional complications in these patients [8]. In addition, blood glucose levels can fluctuate considerably, with MBS patients being particularly susceptible to hypoglycemic episodes, especially following gastric bypass surgery [9]. In patients after MBS, religious fasting could have adverse metabolic effects, and this could lead to increased morbidity and patient hospitalization [10].

Israel has a diverse population that includes individuals from various religious backgrounds, such as Jews, Muslims, Christians, Druze, and others [11]. Each religious group observes its own holidays and fasting practices, including Ramadan for Muslims, Yom Kippur for Jews, Easter for Christians, and more.

The aim of this study is to present the results of a survey on religious fasting in patients after MBS in Israel. The questionnaire was distributed to and completed by members of the Israeli Society of Metabolic and Bariatric Surgery (ISMBS).

## 2. Materials and Methods

This study was conducted by a team of surgeons from a single center: the Tel Aviv Sourasky Medical Center. An online questionnaire survey was designed and sent via email and social media to members of the Israeli Society of Metabolic and Bariatric Surgery (ISMBS) on 8 October 2024 and remained open for 21 days. Participation was voluntary and could be anonymous. The survey consisted of 23 predefined multiple-choice questions addressing religious fasting in patients after MBS. The questionnaire was divided into three sections: (1) MBS surgeon clinical experience, (2) clinical considerations regarding fasting in MBS patients, and (3) fasting-related complications in MBS patients. The questionnaire was originally written in Hebrew, with an English translation available in the electronic Supplementary Material (Supplementary Table S1). All questions were multiple choice, with some allowing multiple selections and question branching. Responses were recorded and presented as numbers (percentages), with results analyzed descriptively and/or graphically.

All procedures performed in this study were in accordance with the ethical standards of the institutional and/or national health research committee and with the 1964 Helsinki Declaration and its later amendments or comparable ethical standards.

## 3. Results

### 3.1. Respondents

The questionnaire was distributed to members of the Israeli Society of Metabolic and Bariatric Surgery (ISMBS) on 8 October 2024 and remained open for 21 days. The ISMBS has 63 active members, and 37 members (59%) responded. The characteristics of respondents are depicted Table 1. More than two-thirds of respondents (*n* = 25, 67.5%) had more than 10 years of MBS experience, and most respondents (*n* = 20, 54%) perform more than 100 MBS procedures annually. The majority of surgeons reported that the most frequently performed MBS procedure was One Anastomosis Gastric Bypass (OAGB) (*n* = 27, 73%). In contrast, Sleeve Gastrectomy (SG) and Roux-en-Y Gastric Bypass (RYGB) were the most commonly performed procedures for a smaller proportion of surgeons (*n* = 5, 13.5%, and *n* = 4, 10.8%, respectively).

### 3.2. Considerations in Religious Fasting

When asked about permitting religious fasting for patients after MBS, 81.1% (*n* = 30) of respondents stated that they would allow it. The considerations to be taken reported by respondents are shown in Table 2.

Time from MBS: The majority of respondents (*n* = 34, 92%) believe that the time elapsed since MBS is an important factor when considering whether to allow patients to fast. The most frequently selected time interval was at least 12 months post-surgery (*n* = 27, 73%), followed by 6 months as the second most common choice (*n* = 5, 13.5%).

Type of MBS: When asked if the type of MBS influences the decision to permit fasting, the majority of respondents (*n* = 27, 73%) stated that it does not. However, 27% (*n* = 10) indicated that it would, with most noting they would permit fasting after Sleeve Gastrectomy (SG) (*n* = 10, 27%) and Laparoscopic Adjustable Gastric Banding (LAGB) (*n* = 8, 21.6%). Hypo-absorptive procedures were less commonly selected, including Roux-en-Y Gastric Bypass (RYGB) (*n* = 6, 16.2%), One Anastomosis Gastric Bypass (OAGB) (*n* = 6, 16.2%), and Single Anastomosis Duodenal–Ileal Bypass with Sleeve Gastrectomy (SADI-S) (*n* = 6, 16.2%). Additionally, 27% of respondents indicated they would permit fasting after SG sooner than after bypass procedures.

Type of fasting: The majority of respondents indicated that the type of fasting would impact their decision to permit it. Specifically, 59.5% (*n* = 22) would allow a one-time 25 h fast, such as Yom Kippur, but would not permit daily continuous fasting lasting 12 h, such as during Ramadan.

### 3.3. Clinical Considerations

Members were asked whether a basic evaluation, including a clinic visit, checkup, and blood work, should be conducted prior to fasting. The majority of respondents (*n* = 23, 62.2%) agreed that a basic evaluation should be performed. In the case that patients would insist on fasting, most respondents would recommend to at least drink fluids (*n* = 32, 86.5%) and consume proton pump inhibitors (*n* = 11, 29.7%).

**Figure 1:**
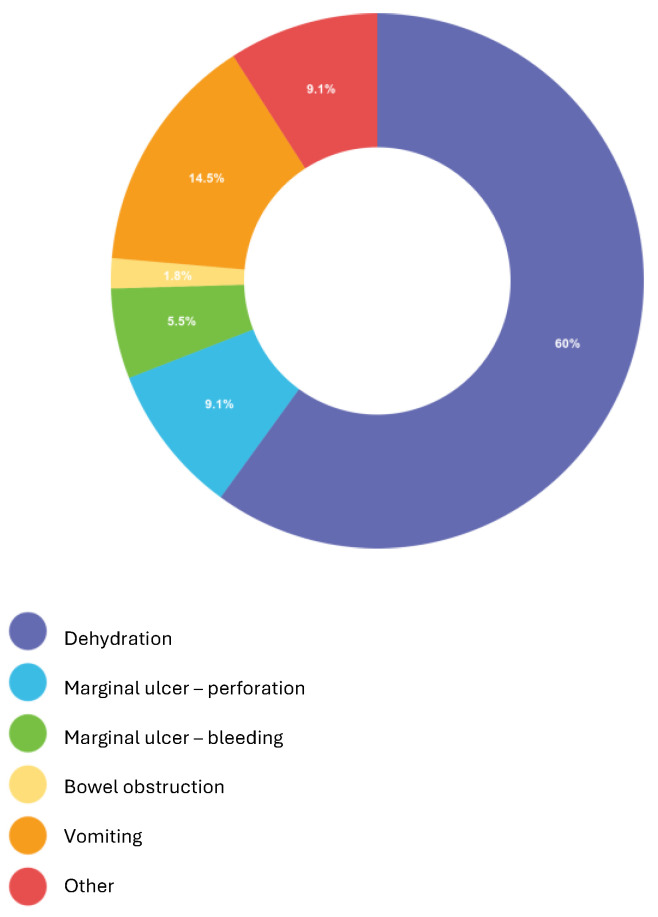
Reported reasons for hospitalization during religious fasting.

Medical care: Twenty-two respondents (59.5%) and twenty-six respondents (70.3%) indicated that there is no increased MBS patient admission to the emergency room (ER) and surgical ward during religious fasting times, respectively. The reason for hospitalization in the ER or surgical ward is shown in Figure 1. Most respondents (*n* = 33, 89%) indicated that they have encountered patients seeking medical care due to dehydration at the time of religious fasting. Most respondents (*n* = 34, 91%) indicated that there are no increased MBS-related emergency surgeries during fasting.

**Figure 2:**
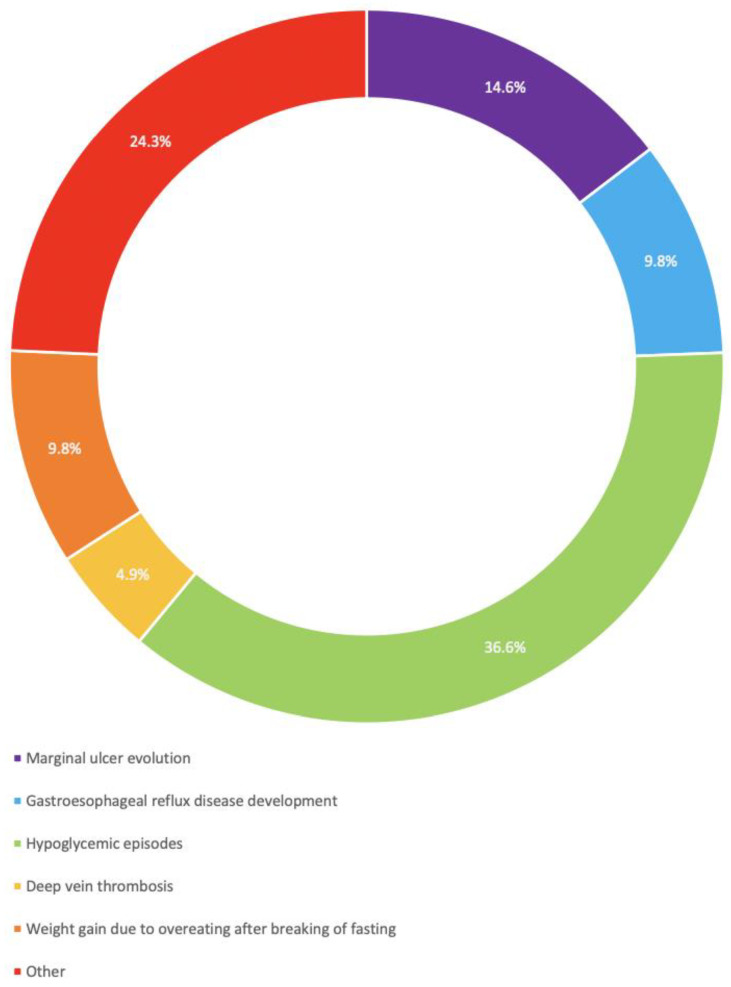
Reported implications of fasting on MBS patients.

Religious fasting risks: The responses for risks of fasting in MBS patients are shown in Figure 2. Fifteen respondents (40.5%) indicated that fasting is associated with hypoglycemic episodes, six respondents (16.2%) related fasting to the evolution of marginal ulcers, four respondents (10.8%) related fasting to the evolution of gastroesophageal reflux and increased weight, and two respondents (5.4%) related fasting with deep vein thrombosis (DVT) development. Ten respondents mentioned other pathologies, including dehydration (*n* = 4) and syncope (*n* = 1), while five out of ten did not specify the pathology.

## 4. Discussion

Fasting as a religious practice is widely observed across various faiths and cultures around the world. Religious fasting has significant effects on human physiology, and the consequences depend on the duration and nature of fasting. Many studies have examined the effects of religious fasting, with a significant focus on intermittent fasting practices like Ramadan. The positive effects reported include improvements in lipid profiles, increased insulin sensitivity, and reduced inflammatory markers [12,13]. Fasting patterns of specific food restrictions may have beneficial effects in reducing cardiovascular disease, enhancing weight loss, and reducing oxidative stress [Bloomer]. Short-term intermittent fasting, such as that observed by Jews during Yom Kippur, may have limited measurable effects due to its brief duration [14]. However, any form of fasting carries potential risks, including dehydration, worsening of chronic medical conditions, and increased anxiety [15,16,17]. This underscores the importance of adopting a personalized approach when advising patients on religious fasting.

Metabolic and bariatric surgeons are often consulted by their patients for medical advice regarding religious fasting. This may involve a complete 25 h fast (no food or drinks) or a prolonged period of time-restricted feeding, where patients fast for half the day, typically lasting up to one month. Patients are usually more concerned the earlier the fasting is following their MBS. There is little evidence supporting an answer to these questions. Most MBS surgeons respond according to their experience. There are no clear guidelines. The absence of a consensus arises from the complexity of balancing religious obligations with medical needs. MBS induces metabolic changes such as nutrient malabsorption, blood sugar alterations, and decreased gastric capacity [18,19]—these could possibly cause negative implications in prolonged fasting. Despite several studies evaluating the safety of fasting in MBS patients, most are limited to small cohorts, questionnaires, and specific populations [20,21,22]. Furthermore, the diversity of fasting practices across religions complicates the development of universal recommendations.

Concerns about dehydration, hypoglycemia, and other medical emergencies often arise. Are these concerns justified? Does the time elapsed since surgery influence these risks? Should counseling differ depending on the type of bariatric procedure performed? In this study, we aim to address these questions based on the experiences of members of the ISMBS.

Kermansaravi et al. [23] published the results of a Modified Delphi Consensus in 2021, involving a committee of 61 renowned metabolic and bariatric surgeons from 24 countries. This consensus focused on the Ramadan fast for Muslim patients after MBS. It recommends that fasting decisions should be made collaboratively between the surgeon, nutritionist, and patient. Most participants advised delaying the first fast until 6–12 months after MBS and suggested that fasting should be avoided if persistent symptoms are present. Given the approximately 7000 MBS procedures performed annually in Israel and the relatively religious population, primarily composed of Jews and Muslims, we believe the experiences of Israeli MBS surgeons are valuable to share.

Most respondents to the questionnaire are experienced MBS surgeons with over 10 years of practice, performing more than 100 procedures annually, primarily OAGB. The majority of surgeons recommended that patients avoid fasting until at least one year after surgery, likely due to the general understanding that recovery takes time, with volume restriction and absorptive physiology gradually improving. Wafa et al. [24] retrospectively analyzed 376 patients who intended to fast during Ramadan within one year of MBS and reported that patients closer to surgery time were more likely to break fasting, present to an emergency department, and experience side effects. They also reported that the overall complication rate was low, suggesting the personalized approach when evaluating fasting in MBS patients. In our survey, for most MBS surgeons, the type of MBS performed does not significantly impact the decision to allow fasting, although some might expect hypo-absorptive procedures to pose a higher risk for dehydration and hypoglycemia. However, opinions are more divided when it comes to different fasting patterns, such as a single 25 h complete fast versus a prolonged daytime fast. It appears that greater caution is applied to prolonged time-restricted feeding (such as during Ramadan) compared to a single day of complete fasting (like Yom Kippur). Prolonged daytime fasting or time-restricted feeding has been well studied in the general population, with minimal effects on weight and fasting glucose levels, averaging a weight loss of up to 2 kg and a decrease of 1.7 mg/dL in blood glucose. These modest changes suggest that concerns about fasting after bariatric surgery may be somewhat exaggerated. Specific fasting patterns may affect MBS patients differently due to variations in the duration, nature, and timing of the fasting period. We added this to the Discussion section.

More than a third of the respondents believe that metabolic and bariatric patients face an increased risk of dehydration during fasting. This concern may be further emphasized in patients undergoing more aggressive hypo-absorptive procedures, where frequent bowel movements and diarrhea are common. It is important to emphasize that this risk could also be high in patients following mainly restrictive procedures such as Gastric Banding, Sleeve Gastrectomy, and Roux-En-Y Gastric Bypass. It is generally agreed that any type of MBS poses patients to a risk of dehydration [25].

The importance of continuing proton pump inhibitors during fasting should be emphasized. This is due to increased concerns about the development of a marginal ulcer during extended periods without food intake. In a retrospective analysis by Kocakusak et al. [26], it was found that peptic ulcer perforation during religious fasting is significantly high, especially in men. Conversely, Roy et al. [27] published a systematic review of the literature and concluded that there is no relationship between religious fasting to H. pylori-induced peptic ulcers, despite the finding of gastric environment remodeling and increased H. pylori colonization. These studies address peptic ulcers; there are no similar studies evaluating marginal ulcers after MBS that could possibly result in different outcomes.

Based on this questionnaire, the most frequent hospitalization reason is dehydration, and there is no increase in emergent surgeries related to MBS. In an international survey on MBS complications during Ramadan fasting, Kermansaravi et al. [23] shared the experiences of MBS surgeons regarding complications in patients during Ramadan. The study included 132 patients, with complications occurring an average of 14.18 months after MBS. The most common issues reported were upper GI symptoms, followed by dehydration, marginal ulcers, and dumping syndrome. Six patients (4.5%) required surgical intervention for their complications. In an interesting retrospective cohort study by Tat et al. [28], it was shown that there are no significant differences in perioperative outcomes, including emergency department visits, readmission rates, reoperations, and complications within 30 days among patients who undergo MBS before or during Ramadan and at times distant to Ramadan.

As mentioned before, there are no clear guidelines on religious fasting after MBS. Interestingly, a review study was reported by a group from the American Society of Metabolic and Bariatric Surgery (ASMBS) on religious fasting after surgery [29]. This study emphasizes the importance of individual assessments, considering the type of surgery, time since surgery, and potential risks like dehydration and malnutrition. It also highlights the need for collaboration between patients and physicians to ensure safe fasting practices while respecting religious commitments. There are no studies evaluating specifically the effect of Yom Kippur fasting on MBS patients, and a common practice in Jewish tradition is to consult with a Rabbi [30]. We hope the results of this survey could be of aid for physicians and MBS patients when considering religious fasting.

This report has several limitations that must be emphasized. The responses to this questionnaire rely solely on the respondents’ experience and subjective feeling. While most participants are experienced MBS surgeons, these data only reflect their opinions. Correlations linking clinical measurements to self-reported features would significantly improve the study’s results. In addition, this questionnaire involved only ISMBS members, and the number of responders is relatively small, which limits the generalizability of the findings. Expanding the survey to include a larger and more diverse group of clinicians—such as dietitians, family physicians, and other specialists involved in MBS patient care—would enhance the comprehensiveness and generalizability of the findings. Moreover, these findings are more specific to Jewish fasting traditions and may not be generalizable to other fasting practices. The findings of this study should be interpreted with caution due to these limitations, and larger-scale studies are needed. Although there have been previous publications and recommendations regarding fasting during Ramadan, this is the first time these questions have been raised in relation to Yom Kippur fasting.

Further studies are required to explore fasting behaviors in patients from diverse religious backgrounds. Studies relating to patients’ perspectives on fasting and adherence to physician instruction could add insightful data.

## 5. Conclusions

In conclusion, these national survey results emphasize the variations in MBS surgeons’ opinions regarding religious fasting after MBS. Despite these differences, there were still many similarities in the responses, such as timing and fasting permission. We believe that the data presented in this survey could aid patients and clinicians regarding the right practice of religious fasting after MBS.

## Figures and Tables

**Table 1:** Characteristics of respondents.

Characteristic	Number (Percent)
Years of MBS Experience	
<3	3 (8.1%)
3–10	9 (24.3%)
>10	25 (67.6%)
Volume of MBS Procedures per Year	
≤50	10 (27%)
50–100	7 (18.9%)
100–200	8 (21.6%)
>200	12 (32.4%)
Most Commonly Performed MBS	
OAGB	27 (73%)
SG	5 (13.5%)
RYGB	4 (10.8%)
Other	1 (2.7%)
Characteristic	Number (Percent)
Years of MBS Experience	
<3	3 (8.1%)
3–10	9 (24.3%)
>10	25 (67.6%)
Volume of MBS Procedures per Year	
≤50	10 (27%)
50–100	7 (18.9%)
100–200	8 (21.6%)
>200	12 (32.4%)
Most Commonly Performed MBS	
OAGB	27 (73%)
SG	5 (13.5%)
RYGB	4 (10.8%)
Other	1 (2.7%)

MBS—Metabolic and Bariatric Surgery. OAGB—One Anastomosis Gastric Bypass. SG—Sleeve Gastrectomy. RYGB—Roux-en-Y Gastric Bypass.

**Table 2:** Religious fasting considerations reported by respondents.

Question	Response
Do you think that religious fasting should be influenced by time from MBS?	Yes—34 (92%) No—3 (8%)
In case the answer was yes, when would you allow religious fasting for patients after MBS?	1 month—0 (0%) 3 months—2 (5.4%) 6 months—5 (13.5%) 12 months—27 (73%)
Do you think that religious fasting should be influenced by type of MBS?	Yes—10 (27%) No—27 (73%)
In case the answer was yes, after which MBS would you allow religious fasting? (multiple answers)	SG—10 (27%) RYGB—6 (16.2%) OAGB—6 (16.2%) LAGB—8 (21.6%) SADI-S—6 (16.2%)
In your opinion is there a difference between one time 25 h fasting (such as Yom Kippur) to continuous 12 h fasting for a 1 month (such as Ramadan)?	Yes—22 (59.5%) No—15 (40.5%)

MBS—Metabolic and Bariatric Surgery; SG—Sleeve Gastrectomy; RYGB—Roux-en-Y Gastric Bypass; OAGB—One Anastomosis Gastric Bypass; LAGB—Laparoscopic Adjustable Gastric Band; SADI-S—Single Anastomosis Duodenal–Ileal Bypass with Sleeve Gastrectomy.

## Data Availability

The raw data supporting the conclusions of this article will be made available by the authors on request.

## Supplementary Materials

The following supporting information can be downloaded at: https://www.mdpi.com/article/10.3390/medicina60122058/s1, Table S1: Questionnaire on religious fasting considerations for ISMBS members.
