# Supplementary material for: Religious Fasting Following Metabolic and Bariatric Surgery (MBS): Insights from Jewish Practices in Israel
Source: Medicina (Kaunas). 2024 Dec 13;60(12):2058. doi: 10.3390/medicina60122058 (PMC11679284; doi:10.3390/medicina60122058)
Supplement: Supplementary file 1 [file medicina-60-02058-s001.zip › medicina-3350718-supplementary.pdf]

Supplementary Table S1 – Questionnaire on religious fasting considerations for ISMBS members

Question	Answer
1.How many years do you practice bariatric surgery?	a. <3 years<br>b. 3-10 years<br>c. >10 years
2. How many bariatric procedures do you perform annually?	a. up to 50<br>b. 50-100<br>c. 100-200<br>d. >200
3. Which bariatric procedure do you perform mostly?	a. Sleeve Gastrectomy<br>b. One Anastomosis Gastric Bypass<br>c. Roux-en-Y Gastric Bypass<br>d. Gastric Band<br>e. Other
4. In general, do you think it is possible to fast after MBS	a. Yes<br>b. No
5. Does the time interval from MBS affect your decision in permitting patient fasting?	a. Yes<br>b. No
6. If you answer is yes, how long form MBS do you permit fasting?	a. 1 month<br>b. 3 months<br>c. 6 months<br>d. > 1 year<br>e. other
7. Do you think fasting depends on type of MBS?	a. Yes<br>b. No
8. Which type of MBS affects your decision on permitting fasting?	a. I will allow fasting earlier after sleeve gastrectomy<br>b. I will allow fasting earlier after gastric bypass<br>c. No difference
9. Following which surgery will you allow fasting? (multiple choice)	a. Sleeve Gastrectomy<br>b.Roux-en-Y Gastric Bypass<br>c. One Anastomosis Gastric Bypass<br>d. Gastric Band<br>e. Single Anastomosis Duodeno-Ileal Bypass<br>f. other
10. Do you think there is a difference between one day fasting (such as Yom Kippur) to continuous approximately 12 hour fasting daily for a month?(such as Ramadan) ?	a. Yes<br>b. No
11. If yes, what is the difference?	a. I will allow fasting for one day<br>b. I will allow fasting in continuous 12 hour fasting

	c. I will allow fluid drinking at least in both<br>d. I will allow fluid drinking at least in one day fasting<br>e. I will allow fluid drinking at least in continuous fasting
12. Do you think a routine patient evaluation should be taken prior to permitting fasting?	a. Yes<br>b. No
13. For patients who insist fasting, what do you recommend?	a. drink fluids during fasting<br>b. proton pump inhibitor consumption<br>c. thromboprophylaxis<br>d. vitamin supplements<br>e. other
14. Do you think there is increased patient admission in religious fasting periods?	a. Yes<br>b. No
15. If yes, which type of fasting did you encounter during the fasting periods?	a. One day fasting (Yom Kippur)<br>b. Continuous 12 hour fasting (Ramadan)<br>c. Other
16. Which type of emergency room admissions do you think increase during fasting periods?	a. Dehydration<br>b. Marginal ulcer perforation<br>c. Marginal ulcer bleeding<br>d. Small bowel obstruction<br>e. Vomiting<br>f. Other
17. Which type of emergency room admissions did you encounter during fasting periods?	a. Dehydration<br>b. Marginal ulcer perforation<br>c. Marginal ulcer bleeding<br>d. Small bowel obstruction<br>e. Vomiting<br>f. Other
18. Do you think there are more surgical ward admissions of bariatric patients during fasting periods?	a. Yes<br>b. No.
19. Do you think there are more bariatric surgical emergencies during fasting periods?	a. Yes<br>b. No.
20. Do you think patients can skip chronic medications and supplements during fasting periods?	a. Yes<br>b. No<br>c. Depends on time from surgery
21. If your answer was "depends on time from surgery", when would you permit?	a. 1 month<br>b. 3 months<br>c. 6 months<br>d. > 1 year
22. What do you think are the implications of fasting on bariatric patients?	a. Marginal ulcer evolution<br>b. Gastroesophageal reflux development<br>c. Hypoglycemic episodes<br>d. Deep Vein Thrombosis

	e. Weight gain resulting from overeating after breaking a fast<br>f. Other
23. If you would like to be a collaborative author please provide your full name	
24. If you would like to be a collaborative author please provide your email address	
